# Model of care and risk factors for poor outcomes in patients on multi-drug resistant tuberculosis treatment at two facilities in eSwatini (formerly Swaziland), 2011–2013

**DOI:** 10.1371/journal.pone.0205601

**Published:** 2018-10-17

**Authors:** M. Verdecchia, K. Keus, S. Blankley, D. Vambe, C. Ssonko, T. Piening, E. C. Casas

**Affiliations:** 1 Medecins Sans Frontieres, OCA-Swaziland, Lomalanga building, Manzini, eSwatini; 2 National TB control programme, Manzini, eSwatini; 3 Medecins Sans Frontieres, The Manson Unit, Lower Ground Floor, Chancery Exchange, London, United Kingdom; 4 Medecins Sans Frontieres, OCA-Berlin, Berlin, Germany; 5 Medecins Sans Frontieres, Southern Africa Medical Unit, Observatory, Cape Town, South Africa; University of Cape Town, SOUTH AFRICA

## Abstract

**Introduction:**

Since 2011 Médecins sans Frontières together with the eSwatini Ministry of Health have been managing patients with multi-drug resistant tuberculosis (MDR-TB) at Matsapha and Mankayane in Manzini region. This analysis describes the model of care and outcomes of patients receiving a 20 months MDR-TB treatment regimen between 2011 and 2013.

**Method:**

We conducted a retrospective observational cohort study of MDR-TB patients enrolled for treatment between May 2011 and December 2013. An extensive package of psychological care and socio-economic incentives were provided including psychological support, paid treatment supporters, transport fees and a monthly food package. Baseline demographic details and treatment outcomes were recorded and for HIV positive patient’s univariate analysis as well as a cox regression hazard model were undertaken to assess risk factors for unfavorable outcomes.

**Results:**

From the 174 patients enrolled, 156 (89.7%) were HIV co-infected, 102 (58.6%) were female, median age 33 years old (IQR: 28–42), 55 (31.6%) had a BMI less than 18 and 86 (49.4%) had not been previously treated for any form of TB. Overall cohort outcomes revealed a 75.3% treatment success rate, 21.3% mortality rate, 0.6% failure and 0.6% lost to follow-up rate. In the adjusted multivariate analysis, low BMI and low CD4 count at treatment initiation were associated with an increased risk of unfavorable outcome.

**Conclusions:**

A model of care that included psychosocial support and patient’s enablers led to a high level of treatment success with a very low lost to follow up rate. Limiting the overall treatment success was a high mortality rate which was associated with advanced HIV and a low BMI at presentation. These factors will need to be addressed in order to improve upon the overall treatment success rate in future.

## Introduction

Multi-drug resistant tuberculosis (MDR-TB) is defined as TB resistant to at least both isoniazid and rifampicin[1]. Worldwide in 2016, among the 10.4 million new cases of TB there were an estimated 490,000 cases of MDR-TB[2].

The current treatment regimens for MDR-TB remain complex and challenging for patients, long in duration with many side effects which may require treatment adaptation[3]. Access to timely diagnosis and patient friendly MDR-TB treatment regimens are still high priorities in the fight against MDR-TB[4].

The Kingdom of eSwatini, in southern Africa, had in 2011 the highest adult HIV prevalence in the world at an estimated 26% among 15–49 years old[5]. Alongside this, the burden of TB was also amongst the highest with an estimated TB incidence of 1,320 per 100,000[6], with 75% of these TB cases co-infected with HIV[6]. Compounding this high TB/HIV burden was a high MDR-TB prevalence comprising 7.7% among new cases and 33.9% among previously treated cases of all TB cases in the Kingdom[7]. In 2010 eSwatini started strengthening its HIV/TB and MDR-TB response with scale up of care and treatment and decentralization of services and in particular of TB care. Currently, eSwatini is in the top 30 list of countries with an high HIV/TB burden[2].

A systematic review and meta-analysis of MDR-TB treatment outcomes among adults reported 49.9% success, mortality 38% and loss to follow up 16.1%[8]. eSwatini reported a treatment success rate of 57.7% in 2011[9] To date we are not aware of any study reporting risk factors for poor outcomes of MDR-TB patients undergoing treatment in eSwatini. In the neighboring South African province of Kwazulu Natal (KZN), which shares a similar HIV epidemic to eSwatini, studies have reported treatment success rates between 44% and 63% with high mortality and lost to follow up rates (18% and 21% respectively)[10,11]. A CD4 count of less than 50 cells/mm^3^ was identified as the highest predictor of unfavorable outcome[11]. In different contexts studies have shown that HIV status, anti-retroviral therapy (ART) status, being underweight, low hemoglobin level, occupation and male sex are predictors of unfavorable outcomes for patients undergoing MDR-TB treatment[12–15]. A systematic review analyzing studies published up to 2008 reported that male sex, alcohol abuse, smear positivity, fluoroquinolone or extensively drug-resistance were associated with poor outcome[16], however a significant limitation of this review in terms of its applicability to the eSwatini context is the limited data that was available for HIV positive patients.

Médecins Sans Frontières (MSF) has been working in collaboration with the eSwatini Ministry of Health (MoH) in Manzini region to improve TB diagnosis, scale up and decentralize MDR-TB care in a patient-centered approach and treatment since 2010. We report here the treatment outcomes of a cohort of MDR-TB patients enrolled between 2011 and 2013, identify risk-factors for unfavorable outcomes and describe the model of care used to achieve these results.

## Methods

This is a retrospective observational cohort study using routinely collected data from two TB treatment sites in Manzini region, eSwatini: Matsapha Comprehensive Health Care Center and Mankayane government hospital. Matsapha comprehensive healthcare clinic is a primary healthcare center offering general out-patient care including integrated HIV and TB care. Matshapa is an industrial centre and many of the patients were factory workers without close family networks. Mankayane general hospital is one of three hospitals in eSwatini with facilities for both in and outpatient drug resistant TB care and HIV integrated care. Mankayane was located in a rural area attracting a rural and stable population with patients having to travel long distances to access health care clinics. Access to TB and HIV diagnosis and treatment is free of charge in both health units.

Patients were enrolled in the study if they had a diagnosis of MDR-TB and started treatment between May 2011 and December 2013. Patients with confirmed extensive drug resistance (XDR-TB) or transferred from any other facility at any point after initiation of MDR-TB treatment were excluded from the study. XDR-TB is defined as resistance to any fluoroquinolone and to at least one of three second-line injectable drugs (capreomycin, kanamycin or amikacin), in addition to rifampicin and isoniazid[1].

Entry points to the drug resistant TB services for patients at these two facilities were through referral from other healthcare facilities, self-referral or through systematic clinical screening at Mankayane or Matsapha facility.

Diagnosis of MDR-TB was based on either phenotypic drug-sensitivity testing (DST) or Xpert testing by Cepheid. In the absence of confirmatory phenotypic or molecular results a presumptive diagnosis of MDR-TB could be made in patients who were at risk of MDR-TB such as previous exposure to first line drugs, failing first line treatment, household or close contacts of confirmed MDR-TB patients and if the patients had compatible clinical and/or radiological findings. Data on resistance to second line drugs was very limited as there was little access to second line DST during this time in eSwatini.

Patients were initiated on treatment by a doctor following eSwatini drug resistant TB guidelines[17]. The treatment consisted of 2 Phases, the intensive phase lasted a minimum of 6 months comprising at least 4 oral drugs (Levofloxacin, ethionamide, terizidone or cycloserine, pyrazinamide and with or without p-aminosalicylic acid (PAS)) and a daily injectable agent (kanamycin or amikacin). The continuation phase lasted a further 12–16 months and consisted of the oral drugs with the exception of the injectable drug. The continuation phase started after the patient had undergone at least 6 months of intensive phase and had at least 2 negative cultures 28 days apart.

HIV care was according to the eSwatini HIV guidelines at the time[18]. HIV co-infected MDR-TB patients not yet on ART were aimed to be initiated on ART between 2 and 8 weeks after TB treatment. Initiation was irrespective of CD4 cell count and patients were only initiated on ART if they were tolerating the TB medications.

The data was collected as part of the routine monitoring and evaluation and therefore we were exempted of written patients consent.

### Model of care

Patients in need of MDR-TB treatment, on the day of diagnosis, received a clinical assessment that included electro cardiogram, audiometry and chest x-ray. On this occasion a full explanation of the disease was given to the patients by a nurse. Subsequently a doctor would perform a routine visit. In the same day an adherence officer delivered patients education on MDR-TB and explained to the patients what package of patient enablers they were entitled to receive. The package included psychosocial support, a food package and transport money. A financial incentive was provided to the treatment supporters who accompanied patients to clinical appointments and had the responsibility to supervise their allocated patient daily observed therapy (DOT). Patients also received an initial psychosocial assessment to understand potential barriers to treatment adherence (e.g. religious beliefs, lack of social and/or family support, use of alcohol or drugs). Soon after, a home viscliait by the outreach team, comprising of a nurse and a psychosocial support officer, was performed to assess barriers to treatment adherence and early identification of any potential social or psychological issues. Family members were also educated on MDR-TB to minimize risk of transmission at home, the treatment supporter received training and contact tracing was performed. On a case by case basis home structural interventions were undertaken in order to improve ventilation or to create a separate living space for the patient to enable an ambulatory home-based care approach to treatment. Monthly visits were performed by the outreach team.

MDR-TB treatment was aimed to be initiated on an ambulatory basis unless patients presented with serious medical conditions that would require hospitalization. Patients received an initial 2-week drug supply and subsequently a 4 weeks supply. Daily drug intake was directly observed by a treatment supporter at the home of the patient.

Clinical follow up consultations to monitor side effects and response to treatment were done on a monthly basis by a doctor at each clinic although in case of clinical complications more frequent follow up could be undertaken if required.

In case of identification of particular psychosocial issues during treatment the patient situation was discussed by the medical team including the psychosocial supervisor. Patients were provided with counselling and when the issues were psychosocial practical interventions included mediation during family meetings or with landlords for housing disputes. Depression treatment was provided when needed.

### Data analysis

Data was collected as part of the routine clinical evaluation of patients. Patient records were kept in a paper based clinic held record; data from these forms was entered into dedicated data collection tool for MDR-TB (Koch’6). Both the paper based and the electronic records were accessed for the purposes of this analysis. Anonymized data was extracted, cleaned and extensively verified for accuracy by both the research team and data clerks at the healthcare facilities. Statistical analysis was done using Stata software (v12.1, Texas Co.). Univariate analysis was undertaken using Chi-square statistical testing. A cox regression hazard model was constructed to determine risk factors predicting unfavorable outcomes among HIV positive patients. Kaplan–Meier estimates were used to describe the cumulative probability of culture conversion and progression to unfavorable outcomes for HIV positive patients.

This study was approved by the eSwatini Ethical Review board on 7^th^ September 2017 and fulfilled the exemption criteria set by the Médecins Sans Frontières Ethical Review Board for a posteriori analyses of routinely collected clinical data, and therefore did not require full MSF ethical review board review or patient written consent. It was conducted with permission from the Medical Director of the MSF Operational Centre Amsterdam.

## Results

**Fig 1:**
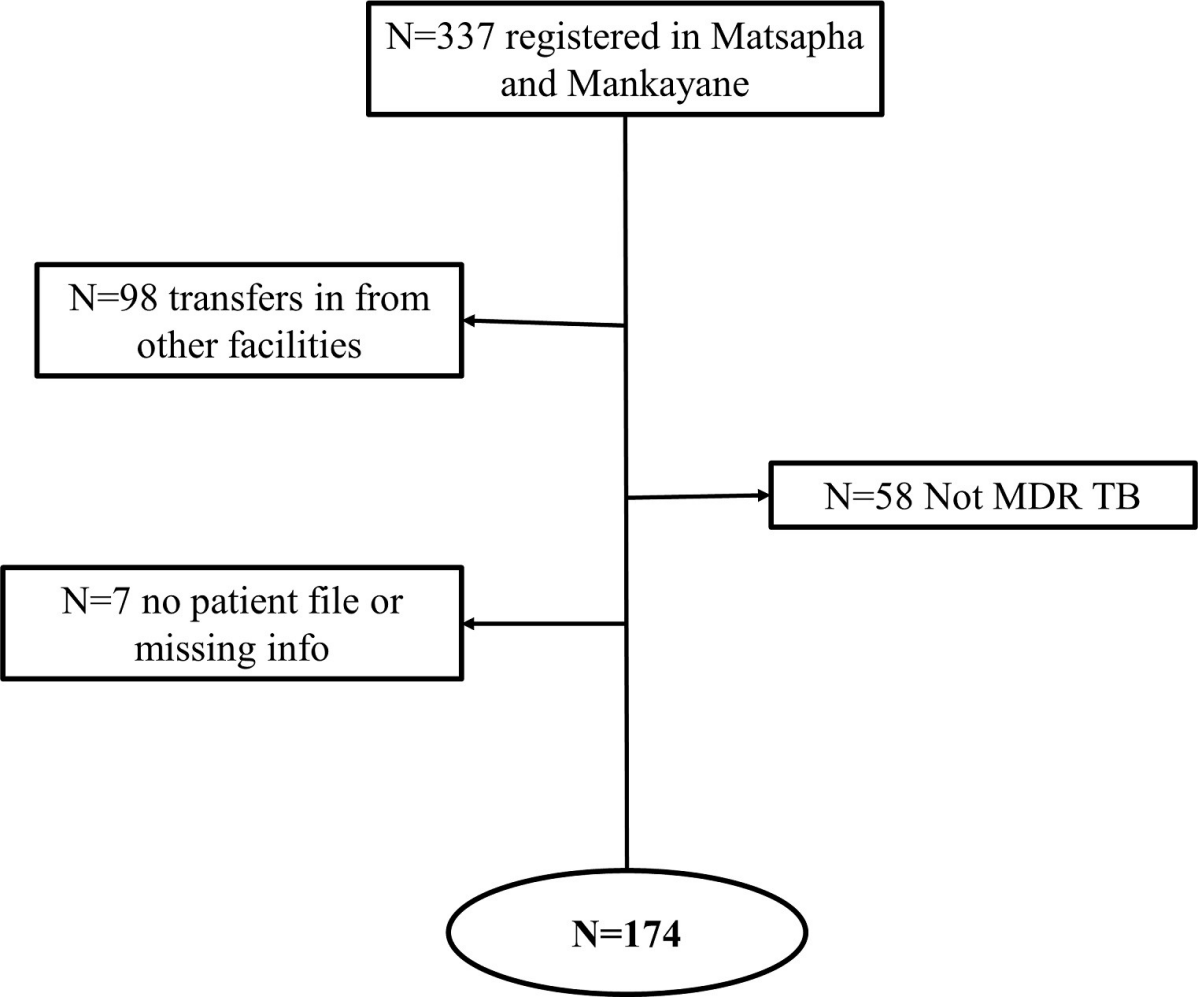
Patients inclusion flow chart.

A total of 337 Drug Resistant TB patients were registered between 2011 and 2013 of which 98 were excluded from this study as they were initiated on MDR-TB treatment at another facility, 58 were not classified as MDR-TB by our diagnostic criteria, and 7 were excluded due to lack of accurate information. A total of 174 MDR-TB patients were included in the final analysis (Fig 1). Of them, 156 (89.7%) had HIV co-infection, 102 (58.6%) were women, 55 (31.6%) had a BMI less than 18 and the median age of the cohort was 33 years old (IQR 28–42). 86 (49.4%) patients were registered as new TB cases. Among 147 HIV positive patients for whom there was complete HIV related information, 47 (32%) had a baseline CD4 at TB treatment initiation of less than 100 cells/mm^3^ and of them 25 (53.2%) were already on ART. Of the total number of HIV co-infected patients, 56 (35.9%) were not yet on ART and 49 (31.4%) had been on ART for more than 12 months (Table 1).

**Table 1:** Demographic and key baseline characteristics.

	**N (%)**
**Total included in the analysis**	174
**HIV+**	156 (89.7)
**Female**	102 (58.6)
**Age (median, IQR)**	*33 (28–42)*
<15	8 (4.60)
15–34	89 (51.2)
35–54	68 (38.1)
≥55	9 (5.2)
**Marital status**	
Married	48 (27.6)
Single	71 (40.8)
Divorced	3 (1.7)
Widowed	8 (4.6)
Missing	44 (25.3)
**Employment**	
Employed	72 (41.4)
Unemployed	53 (30.5)
Pensioner	2 (1.2)
Student	8 (4.6)
Self employed	13 (7.5)
Missing	26 (14.9)
**BMI (median, IQR)**	*19.5 (16.8–21.9)*
Underweight (<18.5)	55 (31.6)
Normal (18.5–24)	83 (47.7)
Overweight (25–30)	14 (8.1)
Obese (>30)	5 (2.9)
Missing	17 (9.8)
**Registration group**	
Missing	1 (0.6)
New cases	86 (49.4)
Previously treated with 1L drugs	75 (43.1)
Previously treated with 2L drugs	12 (6.9)
***Among HIV+ patients***	
**CD4 count (10 missing)**	
<50	20 (13.7)
50–99	27 (18.5)
100–349	65 (44.5)
350+	34 (23.3)
**ART status**	
HIV+ not ART	56 (35.9)
HIV+ & ART for <12 months	51 (32.7)
HIV+ & ART for >12 months	49 (31.4)

**Fig 2:**
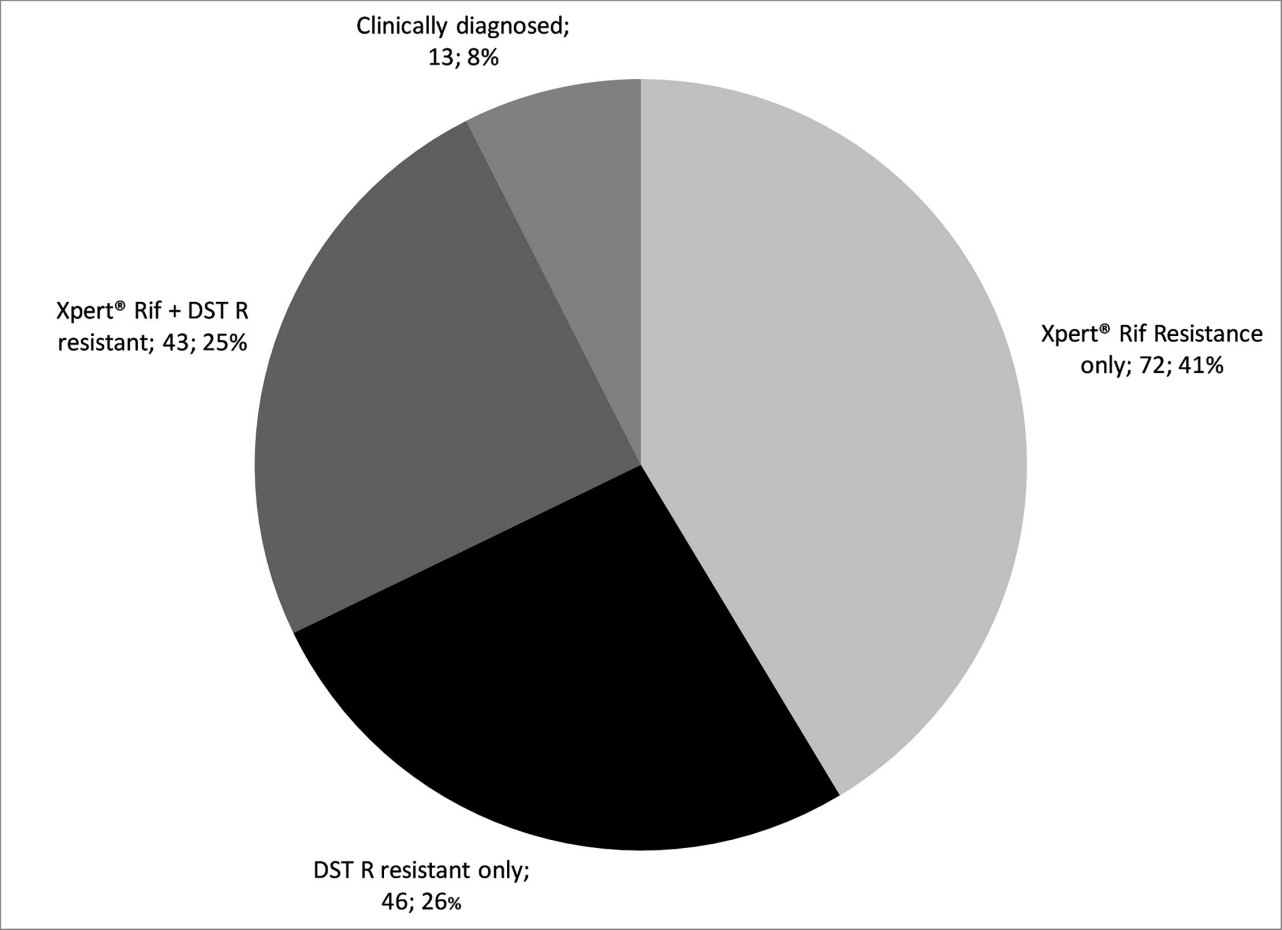
Microbiological diagnosis on patients starting MDR-TB treatment in Matsapha and Mankayane, 2011–20.

A confirmed diagnosis of rifampicin resistance was obtained in 161 (93%) patients with either Xpert, phenotypic DST or both (Fig 2). A presumptive diagnosis was made for 13 (8%) patients. 123 patients had a positive baseline culture and drug sensitivity testing results were available for 90 patients, from which 65 (72.2%) were resistant to all first line drugs (Table 2). A discordant DST and Xpert was obtained in 19 (11.8%) individuals. In 18 out of these 19 cases a rifampicin resistant Mtb strain was identified by DST while the Xpert identified them as rifampicin sensitive. In 1 case DST identified rifampicin sensitive Mycobacterium tuberculosis (Mtb) strain while the Xpert identified rifampicin resistance.

**Table 2:** Baseline bacteriological results N = 174.

Xpert performed	140 (88.5)
MTB+/Rif+	115 (82.1)
**Culture baseline available**	139 (79.3)
positive for M tuberculosis	123/139 (88.5)
**# DST FLD available**	90 (51.7)
**Any resistance**	**N = 90**
Isoniazid	87 (96.7)
Rifampicin	89 (98.9)
Ethambutol	70 (77.8)
Streptomycin	80 (88.9)
**MDR**	
Rif Mono	1 (1.1)
Isoniazid + Rifampicin	5 (5.6)
Isoniazid + Rifampicin + Ethambutol	3 (3.3)
Isoniazid + Rifampicin + Streptomycin	13 (14.4)
Isoniazid + Rifampicin + Streptomycin + Ethambutol	65 (72.2)
**# DST SL available**	14 (8.1)

The overall treatment success rate for the cohort was 75.3% (n = 131) with a mortality rate of 21.3% (n = 37). All the deaths but one occurred among HIV co-infected patients. TB treatment failure was observed in only one patient who was also HIV co-infected, 1 patient was lost to follow up (Table 3). 38% (n = 14) of the deaths occurred within one month of treatment initiation and 22% (n = 8) within the first 15 days.

**Table 3:** End of treatment outcomes for MDR TB patients enrolled between May 2011 and April 2014.

	**total patients**	
	N = 174	
**Outcomes**	**N (%)**	**95% CI**
**Cured**	131 (75.3)	(68.8–81.8)
**Not evaluated**	4 (2.3)	(0.0–4.5)
**LTFU**	1 (0.6)	(-0.6–1.7)
**Died**	37 (21.3)	(15.1–27.4)
**Treatment failure**	1 (0.6)	(-0.6–1.7)

Complete case analysis was used and of the 156 HIV positive patients 135 were included in the multivariate model. In the adjusted multivariate analysis, patients presenting underweight at TB treatment initiation had 4 times (95%CI: 1.6–9.6) the risk of an unfavourable outcome (failures, deaths, lost to follow ups and not evaluated) compared to patients with a normal weight. Being on ART for 12 months or more prior to TB diagnosis reduced the risk of unfavorable outcomes (RR = 0.3, 95%CI: 0.1–0.8) (Table 4).

**Table 4:** Cox Hazard risk model to determine risk factors associated with unfavorable outcome (loss to follow up, treatment failure, death and not evaluated) among HIV positive patients. N = 156.

		**Crude association**		**Adjusted model (N = 135)**	
**Characteristics**	N (%)	HR (95% CI)	P-value	HR (95% CI)	P-value
**Gender**					
Male	62 (39.7)	-		-	
Female	94 (60.3)	0.6 (0.3–1.2)	0.15	0.9 (0.3–2.1)	0.74
Age (linear: 1 year increase)	Median, IQR: 33, (29–41.5)	1.0 (1.0–1.0)	0.27	1.0 (1.0–1.1)	0.26
**Marital status**					
Married	43 (27.6)	-		-	
Single	61 (39.1)	1.3 (0.5–3.0)	0.60		
Other (includes 41 missing,)	52 (33.3)	1.9 (0.8–4.3)	0.14		
**Employment**					
Employed	65 (41.7)	-	-	-	-
Unemployed	46 (29.5)	1.2 (0.5–2.8)	0.61		
Other (includes 26 missing,)	45 (28.9)	2.5 (1.2–5.3)	0.01		
**Treatment history**					
New case	75 (48.1)		-		
1st line drugs	70 (44.9)	0.8 (0.4–1.5)	0.51		
2nd line drugs	11 (7.1)	1.5 (0.5–4.4)	0.44		
**BMI (16 missing)**					
Normal (18.5–24)	76 (54.3)	-	-	-	-
Underweight (<18.5)	48 (34.3)	2.5 (1.2–5.5)	0.02	4.0 (1.6–9.6)	<0.01
Overweight or obese (25+)	16 (11.4)	0.9 (0.2–3.9)	0.85	1.3 (0.3–6.5)	0.75
**ART status baseline (18 missing)**					
Not ART	56 (35.9)	-	-	-	-
ART for > = 12 months	51 (32.7)	0.4 (0.2–0.9)	0.02	0.3 (0.1–0.8)	0.02
ART for <12 months	49 (31.4)	0.7 (0.3–1.4)	0.28	0.7 (0.3–1.7)	0.40
**CD4 count baseline (10 missing)**					
<100	47 (32.2)	-			
100–349	65 (44.5)	0.5 (0.3–1.2)	0.13	0.9 (0.3–2.2)	0.74
350+	34 (23.3)	0.2 (0.2–1.2)	0.12	0.8 (0.3–2.8)	0.79

**Fig 3:**
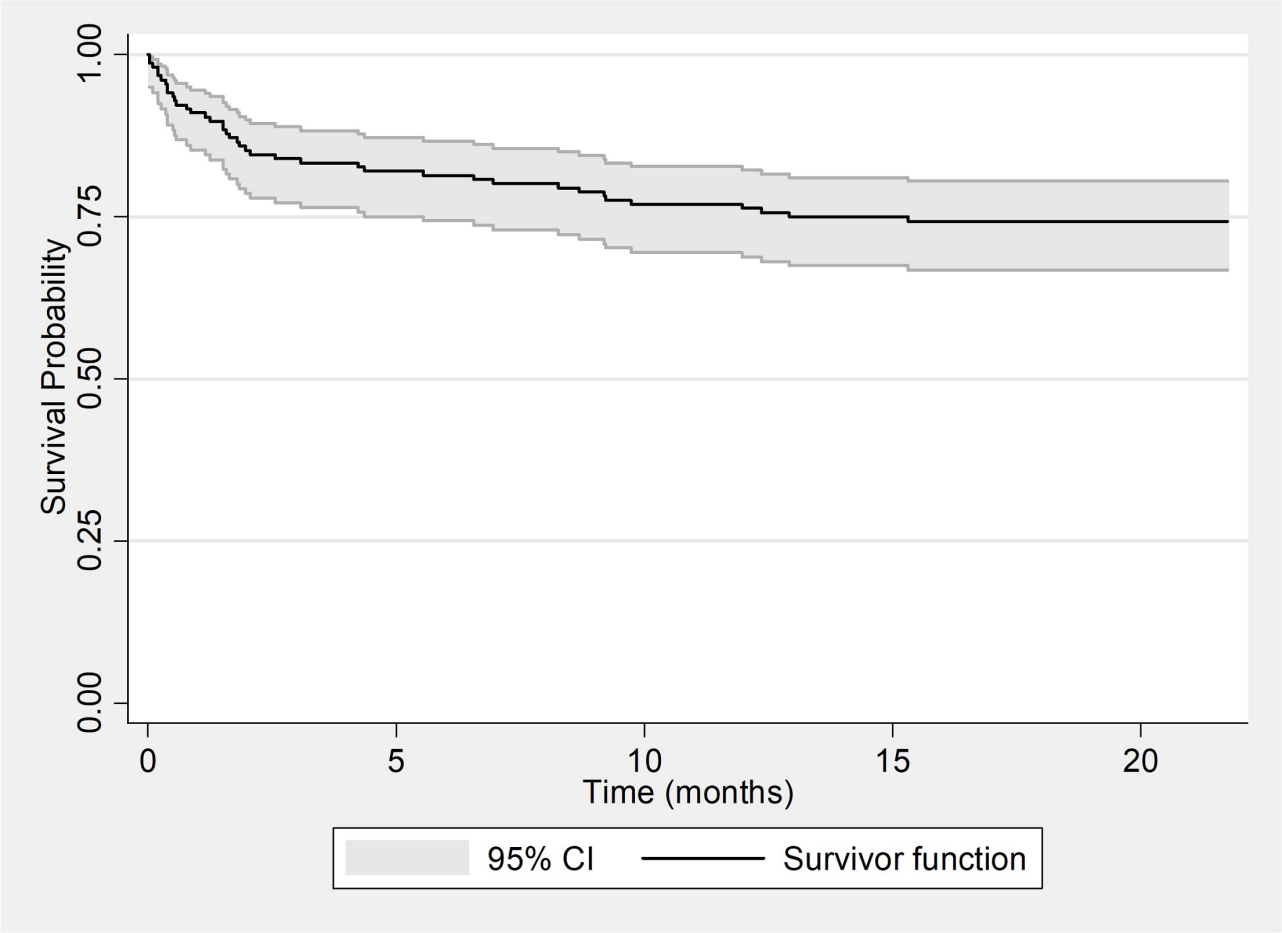
Kaplan-Meier survival estimates for unfavorable outcomes among HIV positive patients.

**Fig 4:**
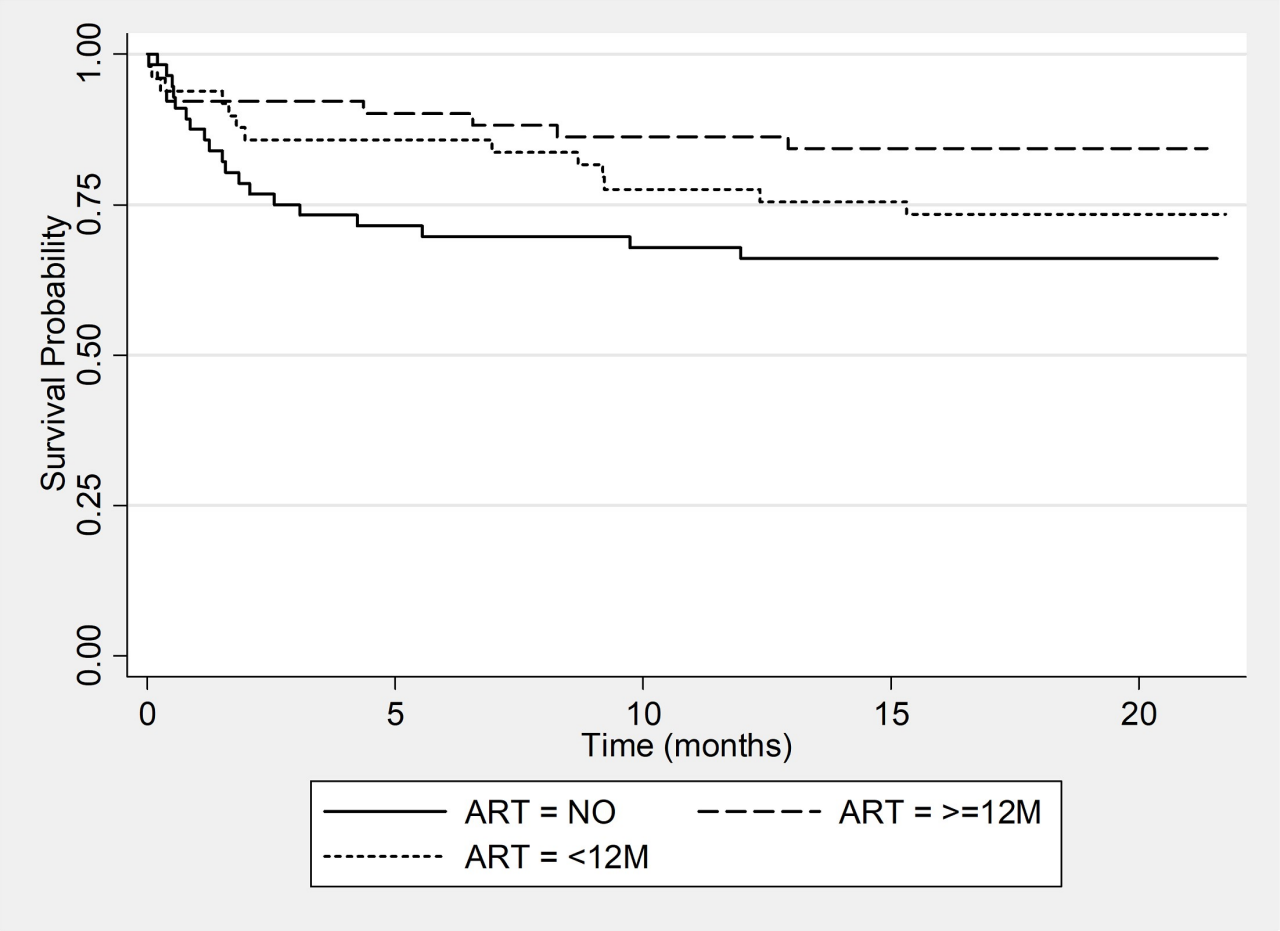
Kaplan-Meier survival estimates for unfavorable outcomes among HIV positive patients by ART status.

**Fig 5:**
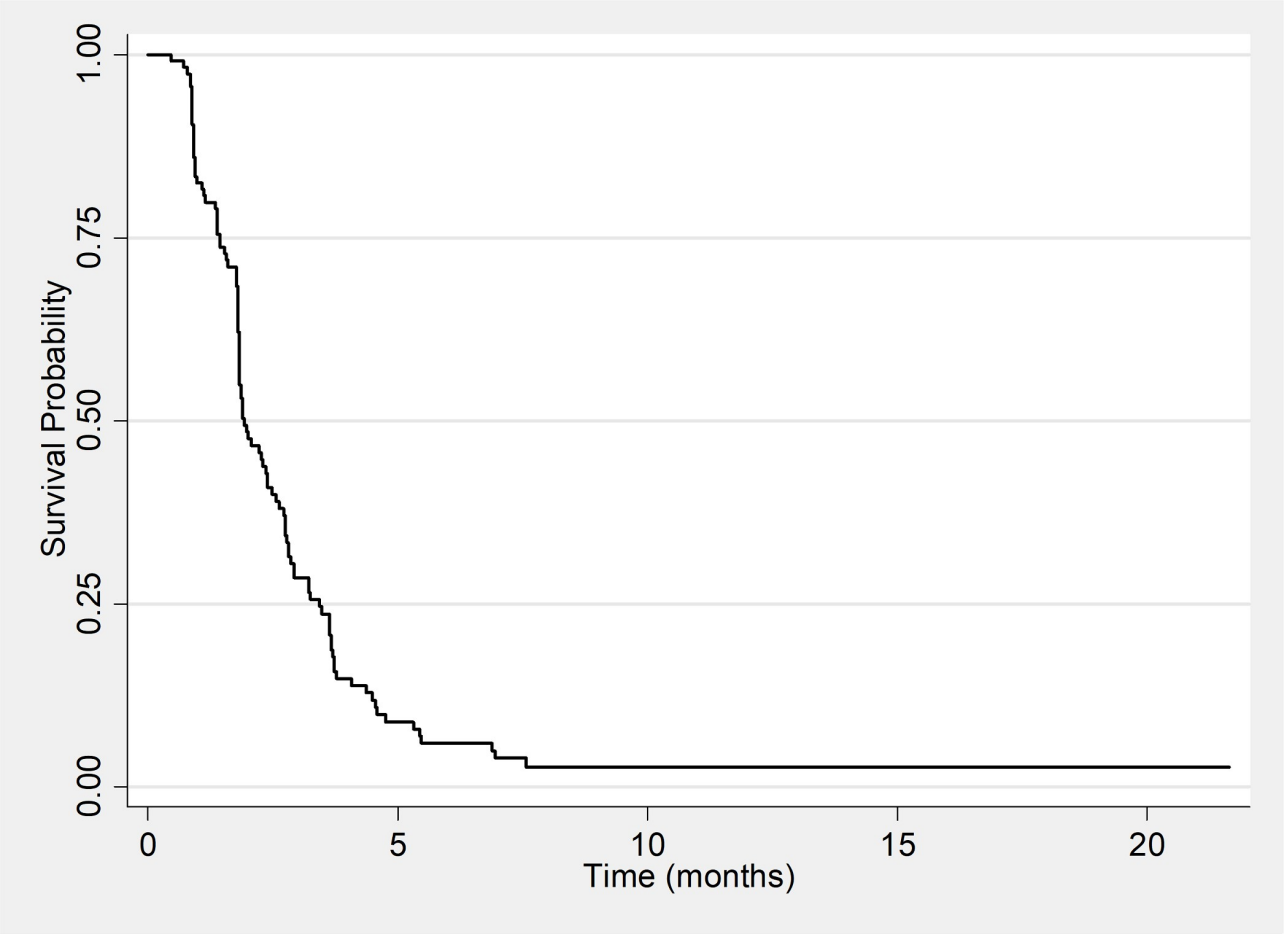
Kaplan-Meier time to culture conversion whole cohort.

The Kaplan-Meier survival curve for the 156 HIV positive patients showed that at 6 months and 12 months of follow up the probability of patients to develop an unfavorable outcome was respectively 19.0% and 24.0% (Fig 3). Fig 4 suggests that patients that were on ARTs for longer than 12 months at initiation of MDR-TB treatment had the highest probabilities of developing an unfavourable outcome compared to patients on ART for less months or not an ART. Of the 123 patients that had a positive culture at baseline the Kaplan Meier curve showed that the probability to culture convert at month 2 and month 4 of treatment was respectively 51.2% and 84.4% (Fig 5).

## Discussion

We report here the treatment outcomes for a cohort of patients enrolled for MDR-TB treatment between 2011 and 2013 at two treatment sites in Manzini region, eSwatini. We show a treatment success rate of 75.3% for a disease that globally had a treatment success rate of 48% in 2013[6].

Studies in the similar high HIV prevalence in South Africa reported treatment success rates of between just 46% and 63%[19,20]. Our treatment success rate is higher than outcomes reported in other settings even with their lower HIV rates.

Contributing to this high treatment success rate is the very low lost to follow up rate in this cohort at just 0.6%, this is much lower than other reported studies[11,12,14–16,19]. This may in part be explained by the high level of support and follow up that patients received under this model of care, an approach which in other studies has been shown to lower lost to follow up rates[21]. This patient centered approach providing treatment and care to patients is designed to take into account the patients circumstances with ambulatory care provided at the medical facility but also home visits if required from the treatment initiation. The psychosocial status of the patients in this study was constantly monitored and patients identified to be in need were closely supported by a psychosocial team.

A systematic review described the psychosocial and economic challenges that MDR-TB patients face with depression, stigma, discrimination, side effects of the drugs causing psychological distress, and the financial constraints due to MDR-TB amongst some of the common issues reported[22]. Our model of care aimed to address these issues in order to improve patients’ adherence to treatment. A qualitative study in Nepal to determine levels of economic and psychosocial problems that MDR-TB patients faced highlighted the need for tailored psycho-social support, especially for the most deprived patients such as those with limited social and financial support[23]. Similar models of care to ours have shown similarly positive results, such as those that have been described in the high HIV burden context in South Africa where support was given to patients in form of transport money, adherence counselling and a home based care approach to allow people in rural settings to adhere to treatment[21]. Another study in South Africa compared hospital based and community based care and showed that patients treated at home were more likely to achieve a successful outcome than those treated in the hospital with a significantly lower lost to follow up among patients that received community based treatment compared to hospital based care[24,25]. In our ambulatory model of care efforts including home rehabilitation projects to ensure infection control standards were undertaken in order to ensure that for those who could receive treatment in their own homes could do so. This home- based care approach may have contributed to the low lost to follow up rate. The Swazi National TB control program has adopted this model of care to treat MDR-TB patients since January 2017.

Mortality in this cohort was seen to occur predominantly in the period just after diagnosis, similar to that found in other studies[26]. The risk for unfavorable outcome in HIV positive patients was increased with low baseline BMI, with a longer time on ART appearing to be protective from poor outcomes. However, the lack of HIV RNA viral load monitoring data did not allow us to relate length of time on ART with successful HIV viral suppression. In our study, CD4 count, which is the best prognostic indicator of mortality in HIV patients, did not appear to be related to mortality or poor outcomes. A better understanding of the causes of mortality in HIV and MDR-TB co-infected population is crucial to design the right interventions. In our study 17% of the HIV co-infected patients were severely immunosuppressed with CD4 counts less than 100 cells/mm^3^ despite already being established on ART. These patients established on ART and developing TB are HIV failure per definition[27] and may have benefited from early switch to second line ART[28]. This underscores the importance and need of strengthening HIV programs and HIV management with early switch of those patients who present with ART failure and severely sick due to MDR-TB[29].

Our analysis carries the limitations related to the retrospective analysis of routine monitoring and evaluation and the description of operational interventions and its inherent risk of information bias. In particular, one of the main limitations in the analysis of prognosis factors was the limited data available on HIV RNA viral load.

In conclusion, while more effective and better tolerated treatment regimens are needed for the management of patients with MDR-TB, this study indicates that a comprehensive model of care including psychosocial support and patient enablers might contribute to satisfactory treatment outcomes in a population with MDR-TB and high rates of HIV co-infection.
